# Production of gene-edited cloned cattle embryos using the CRISPR/EOCas12i system

**DOI:** 10.3389/fgeed.2026.1891100

**Published:** 2026-07-06

**Authors:** Furui Wang, Lei Chen, Yuting Ning, Jiale He, Yinjuan Wang, Lei An, Jianhui Tian, Guangyin Xi

**Affiliations:** College of Animal Science and Technology, Frontiers Science Center for Molecular Design Breeding (MOE), China Agricultural University, Beijing, China

**Keywords:** cattle, MSTN gene, BLG gene, CRISPR/EOCas12i, SCNT

## Abstract

**Introduction:**

The rapid development of genome editing technologies has enabled precise manipulation of livestock genomes for the improvement of production traits such as meat yield and milk quality. Myostatin (*MSTN*) and β-lactoglobulin (*BLG*) are key gene targets for enhancing muscle growth and reducing lactose intolerance, respectively.

**Methods:**

In this study, we employed an optimized CRISPR/EOCas12i system to simultaneously target *MSTN* and *BLG* in bovine fetal fibroblasts (BFFs) using a single plasmid.

**Results:**

T7E1 and Sanger sequencing confirmed efficient editing at multiple target sites, with EOCas12i producing deletions ranging from tens to over 100 bp. Furthermore, no off-target (OT) effects were detected at predicted loci, supporting the high specificity of this system in large animals. Gene-edited single-cell clones (SCCs) were expanded in conditioned medium, and selected double-knockout (DKO) clones served as nuclear donors for somatic cell nuclear transfer (SCNT) to produce *MSTN*/*BLG* double gene-edited cattle embryos.

**Discussion:**

Collectively, this study demonstrates the feasibility of generating *MSTN*/*BLG* double gene-edited cattle embryos using a single CRISPR/EOCas12i plasmid and SCNT, providing a robust platform for multiplex genome editing aimed at improving meat production and milk traits, with potential applications in both agricultural and biomedical research.

## 1 Introduction

Genome editing has emerged as a powerful tool in the field of animal breeding and has been widely applied to improve disease resistance, environmental adaptability, allergenicity reduction, and enhancing productivity (Gim et al., 2022; Lee et al., 2020; Proudfoot et al., 2015). Among the various genome editing approaches, the clustered regularly interspaced short palindromic repeats (CRISPR) system has been extensively utilized for functional genomics studies and genetic improvement. Recently, engineered EOCas12i systems have emerged as promising platforms for multiplexed genome editing (Wang et al., 2025). Notably, EOCas12i retains the advantageous features of Cas12i3, including its relatively small size (∼1045 amino acids) and the ability to autonomously process multiplexed crRNAs (Jin et al., 2025; Zhang et al., 2023), enabling efficient multi-gene editing (Bai et al., 2024) and exhibit simplified protospacer adjacent motif (PAM) requirements (5′-TTN-3′). In addition, EOCas12i demonstrates improved genome editing efficiency, specificity, and enhanced multiplex editing capability in animal cells, often generating larger deletion fragments (Wang et al., 2025). Specifically, the enhanced performance of EOCas12i is attributed to three major engineering strategies. First, crRNA engineering, including stem-loop substitution and direct-repeat optimization, improves target recognition and nuclease activity. Second, Cas12i.3 protein optimization through mammalian codon adaptation and fusion with a 5′exonuclease (T5E) promotes efficient DNA cleavage and facilitates the generation of larger deletions. Third, systematic amino acid engineering within multiple functional domains led to the development of EOCas12i variants with substantially improved editing activity in mammalian cells (Wang et al., 2025). These characteristics highlight its considerable potential for both therapeutic applications and agricultural innovation.

Milk- and meat-related traits have long been major targets in livestock breeding and genetic improvement. Milk is widely recognized as an important source of high-quality proteins and exogenous bioactive peptides. However, the allergenic protein limits its consumption in susceptible individuals. Among these, β-lactoglobulin (BLG) is regarded as one of the major allergens in cow’s milk (Gai et al., 2021; Hattori et al., 2004; Venkatram et al., 2024). Notably, BLG is absent from human breast milk (Kim and Yi, 2020; Lemos et al., 2022), and cow’s milk allergy affects approximately 2%–7% of infants and young children (Hochwallner et al., 2014). To address this issue, numerous studies have attempted to knock out the *BLG* gene in livestock (Song et al., 2015; Tara et al., 2024; Wei et al., 2018; Zhou et al., 2017). In addition to milk traits, muscle growth is another important target in animal breeding. Myostatin (MSTN) is a well-established negative regulator of skeletal muscle development (Aiello et al., 2018). It is predominantly expressed in skeletal muscle, and naturally occurring mutations in *MSTN* have been identified in beef cattle breeds such as Belgian Blue and Piedmontese, leading to the well-known double-muscling phenotype (Kambadur et al., 1997). Based on its pivotal role in muscle development, *MSTN* has also been widely targeted in genome editing studies across multiple farm animal species (Chen et al., 2026; Luo et al., 2014; Wang et al., 2015). Therefore, simultaneous editing of *BLG* and *MSTN* may provide a feasible strategy for the genetic improvement of both milk quality and meat production traits in cattle.

Single-cell clones (SCCs) combined with somatic cell nuclear transfer (SCNT) constitute a classical approach for producing gene-edited animals (Gouveia et al., 2020). SCNT-derived embryo production not only ensures that the genotype of the resulting embryos precisely matches that of the donor cell, but also allows the analysis and verification of edited gene sequences prior to the generation of embryos or animals (Kim et al., 2022; Kim et al., 2019; Moro et al., 2020).

Genome editing of cattle has been extensively explored using CRISPR/Cas9 and related systems, often in combination with SCNT to generate cloned embryos or edited animals from genetically characterized donor cells. Previous studies have successfully generated cattle carrying single-gene modifications targeting economically important traits, including *MSTN* (Gim et al., 2023; Gim et al., 2022; Gim et al., 2022), *BLG* (Tara et al., 2024), *POLLED* (Carlson et al., 2016; Schuster et al., 2020), and Prion Protein (*PRNP*) (Bevacqua et al., 2016). More recently, double-gene editing strategies have also been reported, demonstrating the feasibility of simultaneously modifying multiple loci in cattle (Gim et al., 2023; Rodriguez-Villamil et al., 2024). However, the vast majority of these studies have relied on conventional Cas9-based platforms, the application of Cas12-family nucleases in cattle remains relatively limited. To our knowledge, only a few studies have reported the use of Cas12a or Cas12i for bovine genome engineering, including the generation of hornless cattle through targeted knock-in of the *POLLED* allele (Schuster et al., 2020). Moreover, reports describing multiplex genome editing using Cas12-family systems combined with SCNT in cattle are still scarce. Therefore, the application of the engineered EOCas12i system for multiplex genome editing and cloned embryo production in cattle remains largely unexplored, thereby extending previous studies from conventional Cas9-mediated genome editing toward the application of next-generation engineered Cas12i systems in cattle.

**FIGURE 1:**
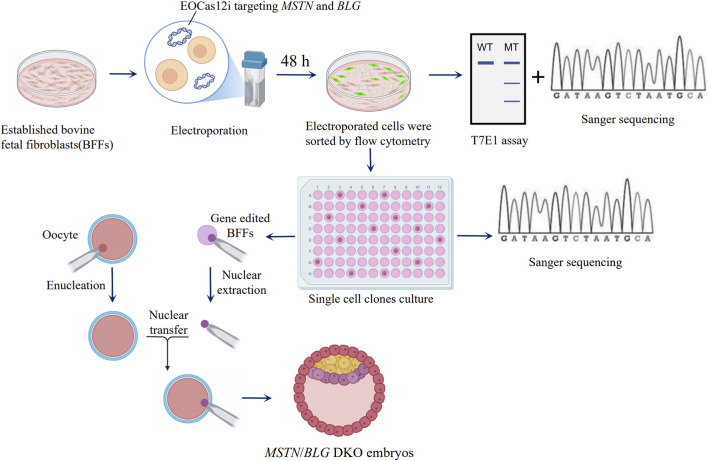
Schematic presentation of the experimental schedule.

In the present study, we employed an optimized CRISPR/EOCas12i system using bovine fetal fibroblasts (BFFs) as the donor cell source to achieve simultaneous knockout of *MSTN* and *BLG* using a single plasmid. Through SCCs expansion, we established three independent *MSTN/BLG* double-knockout (DKO) cell lines, two of them were subsequently used as nuclear donor cells for SCNT, resulting in the successful generation of cattle *MSTN/BLG* DKO blastocysts (Figure 1). This work demonstrates a proof-of-concept pipeline for efficient multiplex genome editing in livestock and provides a foundation for both the production of genetically improved cattle and the development of animal models for biomedical research.

## 2 Methods

### 2.1 Cell culture

Early passage (passages 5–6) BFFs were used for transfection experiments. Cryopreserved BFFs were quickly thawed in a 37 °C water bath and subsequently cultured in KnockOut™ Dulbecco’s modified Eagle’s medium (DMEM #10829018; Gibco, Carlsbad, CA, United States) supplemented with 10% fetal bovine serum (#C04002-500; VivaCell, Denzlingen, Germany), 1% nonessential amino acid (#11140050; Gibco), 1% GlutaMAX Supplement (#35050–061; Gibco) and 1% penicillin-streptomycin (#15140122; Gibco). Cell cultures were maintained in a humidified atmosphere with 5% CO_2_ at 38.5 °C.

### 2.2 Design of crRNAs and their ligation into CRISPR/EOCas12i plasmid

CRISPR RNA (crRNA) targeting bovine *MSTN* and *BLG* was designed using Benchling (https://www.benchling.com/) that showed candidates for the target genome. The corresponding oligonucleotides were commercially synthesized, annealed, and ligated into an CRISPR/EOCas12i plasmid by Genomics (Beijing, China).

### 2.3 Electroporation

For each transfection experiment, 1 × 10^5^ BFFs were seeded into a 10-cm dish and allowed to grow until 60%–70% confluencefor. Cells were trypsinized using (#12563029; Gibco) and resuspened in 100 μL of electroporation buffer EntransterTM-E (#98668; Engreen Biosystem Co.,Ltd., Beijing, China) containing 10 μg of plasmid and transferred to a 0.2 cm gap electroporation cuvette (#1652086, Bio-Rad Laboratories, Hercules, CA, United States). Then, cells were transfected using Nucleofector 2 b Device (LONZA, Basel, Switzerland) with program U-023. Following electroporation, cells were immediately transferred to culture medium in a 10-cm dish and incubated in a humidified atmosphere with 5% CO_2_ at 38.5 °C.

### 2.4 Evaluation of gene editing efficiency

After 48 h post-electroporation, cells were subjected to fluorescence-activated cell sorting (FACS) using a flow cytometer (BD FACSAria™ Fusion, BD Biosciences, United States) to sort green fluorescent protein (GFP)-expressing cells to enrich transfected cells harboring plasmid copies. Genomic DNA from sorted cells was isolated using a commercial kit (#DP304; Tiangen Biotech Co., Ltd. , Beijing, China) and quantified using a spectrophotometer. The polymerase chain reaction (PCR) products generated with 2 × Phanta Max Master Mix (#P525; Vazyme Biotech Co., Ltd., Nanjing, China) on Biometra TAdvanced Thermocycler (Analytik Jena; Jena, Germany). T7 endonuclease I (T7E1) assays were performed to evaluate editing activity under various crRNA. The primers for amplifying target loci in the T7E1 assay are provided in Supplementary Table S1. The annealed samples were then digested with T7E1 (#EN303; Vazyme) for 15 min and separated by electrophoresis on a 2% agarose gel stained with GelRed (#41003; Biotium, Fremont, CA, United States).

We also used a TA cloning method, in which amplified PCR products were cloned into pCE3 Blunt Vectors (#C603; Vazyme) and transformed into *E. coli(Escherichia coli)* cells. Randomly picked *E. coli* colonies were used for plasmid isolation and Sanger sequencing. Sequence data were analyzed for editing events and the editing efficiency was calculated as the number of edited colonies divided by the total number of colonies analyzed.

### 2.5 Prediction of potential off-target (OT) sites

Potential off-target (OT) sites of the crRNAs were predicted using Cas-OFFinder (Bae et al., 2014), The PAM sequence for Cas12i (5′-TTN-3′) and corresponding protospacer sequences were used as input, allowing up to five mismatches. Based on the bovine reference genome (ARS-UCD1.2) (Rosen et al., 2020), Predicted OT sites (Supplementary Table S2) were selected for experimental validation and analyzed by PCR amplification, and T7E1 cleavage assay, as described above.

### 2.6 Generation of single-cell clonal populations and their screening

Prepare conditioned media for single-cell sorts. Collect the BFFs culture media from BFFs during the exponential growth phase (30%–70% confluency) and sterile filter. Mix this with fresh media at a 50/50 ratio and add 5% FBS. After 48 h post-electroporation, use a flow cytometer to sort GFP-expressing cells. The sorted cells are then seeded into 96-well plates coated with 0.1% gelatin (#7903, STEMCELL Technologies, Vancouver, Canada) at single-cell density. After cells have expanded, a portion of the cells is harvested for genomic DNA extraction and PCR, followed by Sanger sequencing (Sangon Biotech, Shanghai, China).

### 2.7 Somatic cell nuclear transfer (SCNT)

The SCCs that underwent *MSTN* and *BLG* double gene editing were selected for SCNT experiments. Ovaries were collected from adult cattle at a slaughterhouse and transported to the laboratory in physiological saline at 30 °C within 4 h. Cumulus–oocyte complexes (COCs) with uniform morphology and compact structure were recovered and washed in M199 medium (Thermo Fisher Scientific, MA, United States). After 16.5 h of *in vitro* maturation, cumulus cells were completely removed using 0.1% hyaluronidase (#H4272, Merck KGaA, Darmstadt, Germany), and oocytes exhibiting uniform cytoplasm and extrusion of the first polar body were selected as enucleated cytoplasmic recipients.

Transgenic BFFs starved in serum-free medium for 2–4 days were trypsinized using TrypLE Select for 4–5 min and transferred into the perivitelline space of the enucleated oocytes. Reconstruction was performed in a solution containing 0.3 M mannitol (MedChemExpress, New Jersey, United States), 0.15 mmol/L Ca^2+^, and 0.15 mmol/L Mg^2+^ for 3–5 min, followed by electrical fusion using a BTX ECM-2001 fusion apparatus. Reconstructed embryos were immediately transferred into M199 medium supplemented with 10% FBS and incubated for 1 h to assess fusion efficiency. Successfully fused embryos were then activated in 5 µM ionomycin (Merck KGaA) for 5 min, followed by incubation in 1.9 mM 6-DMAP (Yeasen Biotech Co., Ltd., Shanghai, China) for 4 h, and subsequently cultured in CR1aa medium containing 5% FBS. Embryo developmental competence was recorded on day 2 (cleavage rates) and day 8 (blastocyst formation rates).

### 2.8 Statistical analysis

Statistical analyses were performed using GraphPad Prism 8.00 software (GraphPad, CA). Differences among groups were assessed using one-way ANOVA. Data are presented as means ± SEM from three independent biological replicates (n = 3). All groups share the same superscript “a”, indicating no significant differences were observed (p ≥ 0.05).

## 3 Results

### 3.1 Screening of efficient crRNAs targeting *MSTN* and *BLG* genes

**FIGURE 2:**
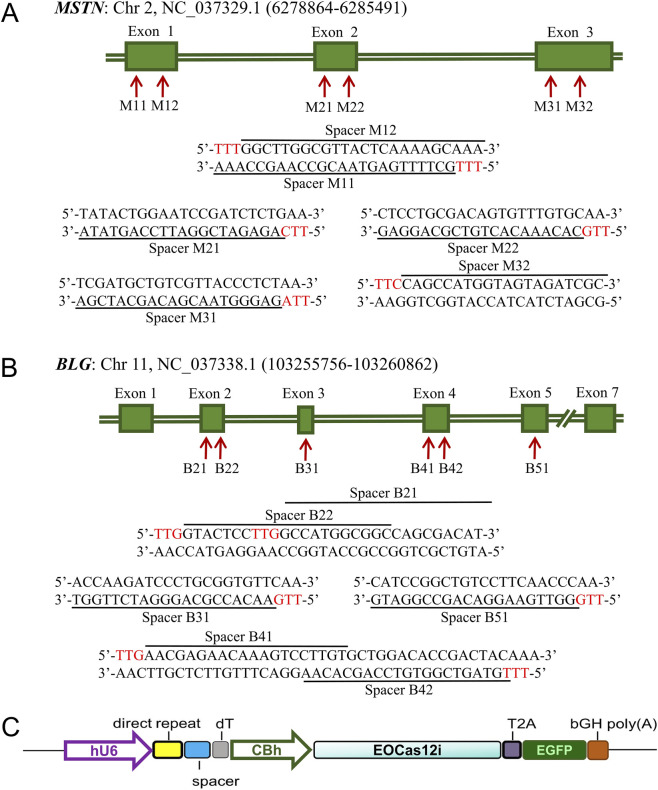
Target sites, sequences, and plasmid map for *MSTN* and *BLG*. **(A,B)** Positions and sequences of target sites in *MSTN* **(A)** and *BLG* **(B)**. Protospacer adjacent motif (PAM) sequences are indicated in red, and the corresponding spacers are underlined. The spacers shown in panels **(A)** and **(B)** are located immediately downstream of the direct repeat. The selected target sites are located within coding exons of both genes, enabling disruption of the protein-coding sequences upon genome editing. **(C)** Schematic representation of the CRISPR/EOCas12i plasmid used in this study.

Due to the distinct editing characteristics of the EOCas12i system compared with Cas9, a total of 12 crRNAs were designed targeting multiple exons of the *MSTN* and *BLG* genes (6 crRNAs for each gene). The target sequences and their genomic locations are shown in Figures 2A,B, and the positions of the spacers within the plasmids are illustrated in Figure 2C.

**FIGURE 3:**
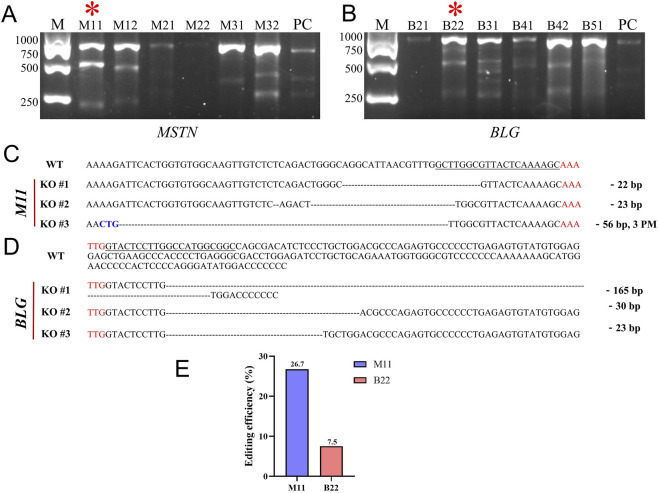
Validation of single knock-out crRNA efficiency by T7E1 assay and Sanger sequencing. **(A,B)** BFFs were transfected with CRISPR/EOCas12i plasmids containing the corresponding spacers. Genomic DNA was isolated, and the *MSTN* **(A)** and *BLG* **(B)** target regions were PCR amplified and subjected to T7E1 cleavage assay. Representative cleavage patterns are shown in the M11 lane of panel **(A)** and the B22 lane of panel **(B)**. In addition to the full-length 700 bp bands, prominent cleavage bands are observed at ∼500 bp and ∼200 bp. M, DNA marker; PC, positive control. **(C)** Sequencing results for crRNA M11 targeting *MSTN*. PAM sequences are highlighted in red, the corresponding spacers are underlined, and deletions are indicated by dashed lines. PM, point mutation. **(D)** Sequencing results for crRNA B22 targeting *BLG*. PAM sequences are highlighted in red, the corresponding spacers are underlined, and deletions are indicated by dashed lines. **(E)** Quantification of editing efficiency of M11 and B22 crRNAs based on TA-cloning and Sanger sequencing analysis. Editing efficiency was calculated as the percentage of edited *Escherichia coli* colonies among total analyzed colonies (M11: 8/30; B22: 3/40).

Each single-KO CRISPR/EOCas12i plasmid was individually transfected into BFFs via electroporation. To assess genome editing efficiency, GFP-positive cells were enriched by FACS, followed by genomic DNA extraction. PCR primers were designed to amplify approximately 700 bp fragments spanning each target site, with the predicted cleavage regions located around 200–500 bp within the amplicons. T7E1 cleavage assay revealed that among the 12 crRNAs tested, M11 and B22 exhibited the highest editing efficiencies, as indicated by the most prominent cleavage bands (∼500 bp and ∼200 bp; Figures 3A,B). To further validate the editing outcomes, PCR amplicons were subjected to TA cloning followed by Sanger sequencing. In the M11 group, 8 out of 30 *E. coli* colonies harbored deletions ranging from 22 to 56 bp, corresponding to an editing efficiency of 26.7% (Figures 3C,E). In the B22 group, 3 out of 40 colonies carried a 165 bp deletion, corresponding to an editing efficiency of 7.5% (Figures 3D,E). Editing efficiency was calculated as the number of edited colonies divided by the total number of colonies analyzed. Based on these results, M11 and B22 were selected for subsequent construction of double-knockout (DKO) plasmids targeting *MSTN* and *BLG*.

### 3.2 *MSTN*/*BLG* double-knockout (DKO) and off-target (OT) analysis

**FIGURE 4:**
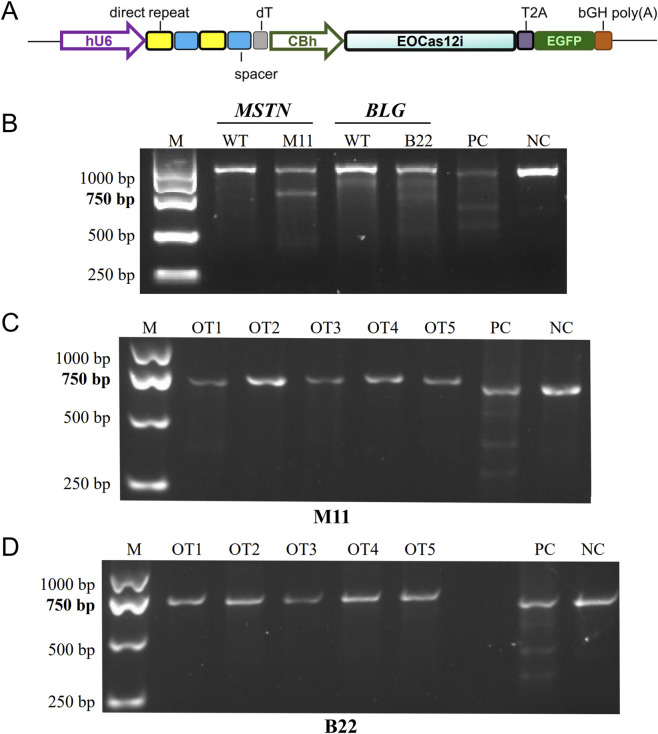
Validation of double KO crRNA efficiency and off-target analysis. **(A)** Schematic representation of the CRISPR/EOCas12i plasmid containing spacers targeting both *MSTN* and *BLG*. **(B)** BFFs were transfected with the plasmid shown in panel **(A)**. Genomic DNA was isolated, and the *MSTN* (M11) and *BLG* (B22) target regions were PCR amplified and subjected to T7E1 cleavage assay. In the wild-type (WT) control, only the full-length 700 bp bands are observed. In the M11 and B22 lanes, in addition to the full-length 700 bp bands, prominent cleavage bands are detected at approximately 500 bp and 200 bp. M, DNA marker; WT, wild type; PC, positive control; NC, negative control. **(C,D)** T7E1 cleavage assays of predicted off-target sites for M11 **(C)** and B22 **(D)** crRNAs. No detectable off-target cleavage was observed. M, DNA marker; PC, positive control; NC, negative control.

M11 and B22 crRNAs were cloned into a single CRISPR/EOCas12i plasmid, with their arrangement is illustrated in Figure 4A. The constructed plasmid was transfected into BFFs by electroporation as described above, followed by sample collection. Consistent with the single-knockout results, T7E1 cleavage assays showed prominent cleavage bands at approximately 500 bp and 200 bp for both the M11 and B22 targets (Figure 4B), indicating that simultaneous editing of *MSTN* and *BLG* can be achieved using a single CRISPR/EOCas12i plasmid. To further assess potential off-target (OT) effects, predicted candidate OT sites were amplified and subjected to T7E1 cleavage assays (Figures 4C,D). No detectable cleavage was observed, suggesting the absence of OT effects under the conditions tested.

### 3.3 Production of gene-edited cloned embryos

**FIGURE 5:**
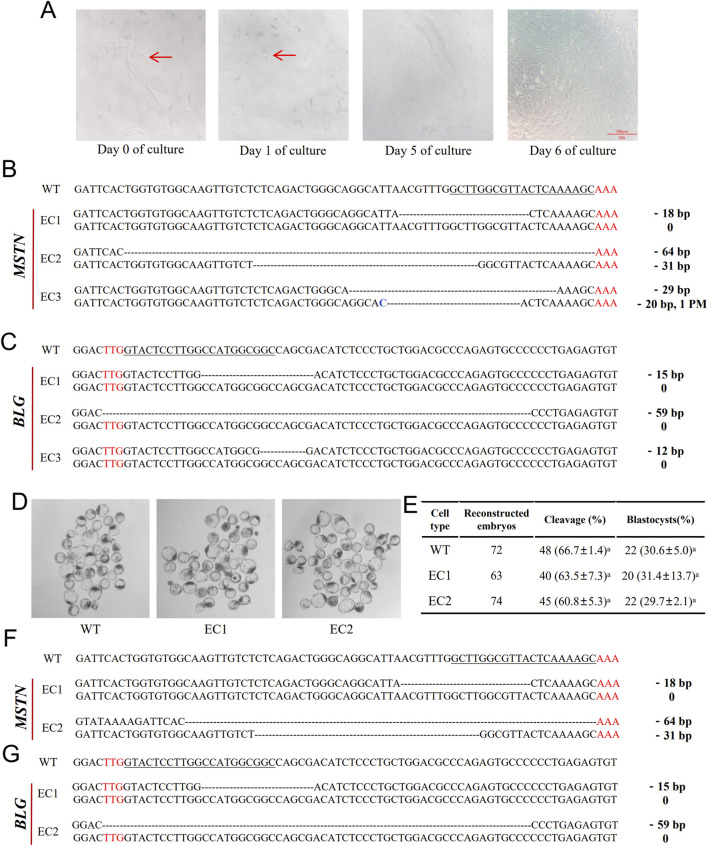
Single cell clones, genotyping, and developmental competence of SCNT embryos derived from *MSTN*/*BLG* gene-edited cells. **(A)** Representative images of single cell clone growth. Red arrows indicate individual cells. **(B,C)** Sanger sequencing results for *MSTN* **(B)** and *BLG* **(C)** of three *MSTN*/*BLG*-edited cell lines, designated EC1, EC2, and EC3. PAM sequences are highlighted in red, the corresponding spacers are underlined, and deletions are indicated by dashed lines, point mutations are highlighted in blue. PM, point mutation. **(D,E)** *In vitro* development of SCNT embryos generated from wild-type (WT), EC1, and EC2 BFFs, showing representative images **(D)** and corresponding developmental rates **(E)**. Data are presented as means ± SEM (n = 3). All groups are marked with the same superscript “a”, indicating no significant differences were observed among groups (p ≥ 0.05, one-way ANOVA). **(F,G)** Sanger sequencing results for *MSTN* **(F)** and *BLG* **(G)** of cloned embryos derived from the EC1 and EC2 donor cell lines. PAM sequences are highlighted in red, the corresponding spacers are underlined, and deletions are indicated by dashed lines.

After confirming that a single CRISPR/EOCas12i plasmid could simultaneously generate DKO events at the *MSTN* and *BLG* loci, GFP-positive cells were enriched by FACS. Sorted cells were plated in 96-well plates at single-cell density and cultured for approximately 6 days, after which colonies were collected for genotyping. Ultimately, three independent *MSTN*/*BLG*-edited single-cell clones (SCCs) with stable proliferation and confirmed genotypes were established and subjected to further characterization (Figure 5A). Sanger sequencing revealed that clone EC1 harbored monoallelic deletions at both *MSTN* and *BLG*, whereas clones EC2 and EC3 carried biallelic mutations at *MSTN* together with monoallelic deletions at *BLG* (Figures 5B,C).

Among these characterized SCCs, EC1 and EC2 were selected as nuclear donors for cloned embryo production via handmade cloning (HMC) (Figure 5D). Analysis of cleavage and blastocyst formation rates indicated that the developmental competence of embryos derived from *MSTN*/*BLG*-edited donor cells was comparable to that of embryos from non-edited donor cells, suggesting that early embryonic development was not obviously affected under the conditions examined (Figure 5E). Furthermore, genotyping of the resulting blastocysts confirmed that their sequences were identical to those of the donor single-cell clones (Figures 5F,G), supporting the fidelity of genome editing and the applicability of this workflow for generating *MSTN*/*BLG* DKO bovine embryos.

## 4 Discussion

With the development of genome editing technologies, the genomes of farm animals have been increasingly manipulated to improve production traits, such as milk yield and meat quality, providing new avenues to address challenges in human nutrition (Bosch et al., 2015; Van Eenennaam, 2018). MSTN, a key negative regulator of skeletal muscle growth, has been a major target for genetic modification to produce animals with enhanced muscularity, and several *MSTN*-edited livestock have been successfully generated in recent years (Selokar et al., 2025). BLG, an important protein involved in milk composition, represents a critical target for mitigating lactose intolerance in susceptible populations. In the present study, we employed the CRISPR/EOCas12i system to simultaneously suppress both *MSTN* and *BLG* expression in BFFs using a single plasmid. Subsequently, SCNT was performed to generate *MSTN*/*BLG* DKO cattle blastocysts, demonstrating the feasibility of multiplex genome editing in livestock using this approach. Furthermore, no OT effects were detected at the predicted sites following T7E1 cleavage assays, confirming the applicability of EOCas12i in large animals.

In genome editing, target-site selection remains a major determinant of editing outcomes, as cleavage efficiency can vary substantially among loci even when the same nuclease is used. Differences in editing efficiencies among target sites have been widely observed in CRISPR-based genome editing. For example, Cas9-mediated editing is often more efficient in regions of open chromatin than in closed chromatin, indicating that chromatin accessibility influences nuclease access and activity (Liu et al., 2019). Systematic analyses across hundreds of genomic loci have also revealed that sequence context and protospacer features correlate with both editing precision and efficiency, suggesting that local sequence determinants contribute to variable editing outcomes (Chakrabarti et al., 2019), locus-specific factors also influence editing efficiency and indel patterns (Jensen et al., 2017). In the present study, *MSTN*-directed crRNAs exhibited a markedly higher success rate (26.7%) than *BLG*-directed crRNAs (7.5%). This trend is also consistent with previous reports showing that the *BLG* locus can be relatively refractory to genome editing. In CRISPR/Cas9-mediated *BLG* editing in buffalo fibroblasts, single sgRNAs showed relatively about 50% editing activity (Tara et al., 2024). In contrast, *MSTN* targeting in livestock species often yields higher individual sgRNA efficiencies, with several studies reporting multiple guides up to 70% editing *in vitro* (Ni et al., 2014; Zhou et al., 2022), suggesting that *MSTN* may be intrinsically more permissive to nuclease activity and/or cellular repair outcomes, while the *BLG* locus likely reflects intrinsic characteristics of the target region. Furthermore, given that the identified mutations occurred within coding regions and included deletion events predicted to disrupt the reading frame and/or protein structure, both *MSTN* and *BLG* protein products are likely to be altered. However, direct validation of protein expression and phenotypic consequences will require further analysis after the birth of edited animals. In addition, although present editing efficiencies are lower than those typically reported for optimized Cas9-based systems, they are consistent with early-stage applications of emerging genome-editing platforms in large animals, where editing efficiency is often highly locus-dependent and system-dependent (Schuster et al., 2020). Importantly, successful genome modification was achieved despite relatively modest editing rates, demonstrating the practical applicability of the engineered EOCas12i system in bovine somatic cells.

Notably, in the present study, EOCas12i generated deletions of several tens to over 100 bp at different sites within two target genes, substantially larger than the typical deletions produced by Cas9, which are usually only a few to a dozen base pairs. This editing pattern is likely attributable to the molecular design of EOCas12i, which incorporates mammalian codon optimization together with fusion of a T5E to enhance cleavage activity and promote end resection (Wang et al., 2025). Such large-fragment deletions may provide practical advantages for functional knockout studies because they increase the probability of disrupting coding sequences and reduce the likelihood of generating partially functional alleles. These frameshift-inducing mutations are strongly predictive of protein loss of function.

Interestingly, during the SCCs expansion process, the growth rate of our SCCs was notably faster than commonly reported, where 10–20 days is typically required to achieve confluency in 96-well plates (Dua et al., 2023). In our system, individual SCCs reached confluence within 6 days. One likely contributor to this enhanced expansion is the use of conditioned medium during cloning. Conditioned medium, which contains secreted growth factors and extracellular matrix components from actively proliferating feeder cells, has been shown to significantly improve the survival, attachment, and proliferation of isolated single cells during clonal selection by recreating a supportive microenvironment. Such supportive effects have been documented in multiple cell types, including primary fibroblasts and stem cells, where conditioned medium markedly increases cloning efficiency and reduces apoptosis compared to standard medium (Caneparo et al., 2020; Liu et al., 2022).

However, the limitation of the current study is that we did not obtain a *BLG* biallelic knockout cell line. This may reflect locus-dependent editing variability at the *BLG* locus, which could be influenced by local chromatin accessibility, GC content, sequence context, or DNA repair preferences, as discussed above. Notably, *BLG* editing has been reported in livestock, indicating that biallelic disruption of this gene is technically achievable (Ni et al., 2014; Sun et al., 2018), although further optimization of crRNA selection, delivery conditions, and clone screening may be required. Further optimization of experimental conditions is also required to improve the genome-editing efficiency of the EOCas12i system in bovine cells. Similar to CRISPR/Cas9, editing outcomes may be influenced by delivery strategy, crRNA design, and target-site accessibility. In addition, the developmental assessment of SCNT embryos in this study was mainly based on cleavage and blastocyst formation rates. Although these results preliminarily suggested that early embryonic development was not obviously impaired, additional embryo-quality indicators, such as total cell number and differentiation of inner cell mass and trophectoderm, should be evaluated in future studies to more comprehensively assess the developmental competence of gene-edited cloned embryos. It is also worth noting that using pluripotent stem cells as nuclear donors may further improve reprogramming efficiency during SCNT (Chen et al., 2026; George et al., 2018; Matoba et al., 2025; Sharma et al., 2011), potentially enhancing the generation of edited embryos. Nevertheless, genome editing progress in cattle has historically lagged behind that in other mammalian species, largely due to challenges such as long gestational periods, single offspring per pregnancy, and the high costs associated with maintaining and manipulating large animals (Gouveia et al., 2020; Thuan et al., 2010).

In conclusion, we successfully demonstrated the production of cattle *MSTN*/*BLG* double gene-edited embryos using the CRISPR/EOCas12i system combined with SCNT. With optimized protocols and advanced genome editing strategies, our long-term goal is to generate genome-edited cattle with enhanced meat production and milk traits that minimize lactose intolerance. Moreover, this study provides a foundation for ensuring the safety and stability of meat and milk derived from genetically modified animals. Importantly, the CRISPR/EOCas12i system in combination with SCNT offers a versatile platform for the simultaneous modification of multiple genes of agricultural and biomedical relevance, including traits associated with disease resistance.

## Data Availability

The original contributions presented in the study are included in the article/Supplementary Material, further inquiries can be directed to the corresponding author.
