# Supplementary material for: Production of gene-edited cloned cattle embryos using the CRISPR/EOCas12i system
Source: Front Genome Ed. 2026 Jul 6;8:1891100. doi: 10.3389/fgeed.2026.1891100 (PMC13381622; doi:10.3389/fgeed.2026.1891100)
Supplement: Supplementary file 1 [file Supplementaryfile1.docx]

Table S1: Primer Sequences for *MSTN* and *BLG*.

| **Primer** | **Sequences (5’-3’)** | **Application** |
| --- | --- | --- |
| M11-F | ATCAGCTCACCCTTGACTGT | PCR |
| M11-R | GTCAGCAGAGTCGTTGCTCT | PCR |
| M12-F | ATCAGCTCACCCTTGACTGT | PCR |
| M12-R | GTCAGCAGAGTCGTTGCTCT | PCR |
| M21-F | TGGAAAGGAAGTAGGCTTCTCA | PCR |
| M21-R | AAGCACAGGAAACTGGTAGTTA | PCR |
| M22-F | TGGAAAGGAAGTAGGCTTCTCA | PCR |
| M22-R | AAGCACAGGAAACTGGTAGTTA | PCR |
| M31-F | ACAGGGGAATCCCTATGGCTA | PCR |
| M31-R | TGTAGCTTGTGCTTAAGTGACTG | PCR |
| M32-F | ACAGGGGAATCCCTATGGCTA | PCR |
| M32-R | TGTAGCTTGTGCTTAAGTGACTG | PCR |
| B21-F | TTCTCCCTGTGTCCTTTGCG | PCR |
| B21-R | ATAAGCAGCCTTGGGTGGAC | PCR |
| B22-F | TTCTCCCTGTGTCCTTTGCG | PCR |
| B22-R | ATAAGCAGCCTTGGGTGGAC | PCR |
| B31-F | CCAACCTTTTCTCCCTGGCT | PCR |
| B31-R | GCGGACAGAGTTCGTCTTCA | PCR |
| B41-F | GGATCCAGAGTTGGGCTTCC | PCR |
| B41-R | AGACAGCTTTGTGCCTGAGT | PCR |
| B42-F | GGATCCAGAGTTGGGCTTCC | PCR |
| B42-R | AGACAGCTTTGTGCCTGAGT | PCR |
| B51-F | GGAGGAAGGGGAGGGAACT | PCR |
| B51-R | CAGGAAGGAGGTGGAGGAGA | PCR |

Table S2: Potential off-target sites and their pirmers.

| **crRNA** | **Off-target** | **Primer sequences (5’-3’)** | **Application** |
| --- | --- | --- | --- |
| M11 | TTTtGCTTTTGAGTAACGCCAAGC | ACTTCATCTGAACTAACGTGGAGAA | PCR |
|  |  | CCTGGACATGCAGCCTGTTTT | PCR |
|  | TTTGCTTTTGAGTAACGCCAAGcC | TAATGTCTGGCTTGGGAAGGG | PCR |
|  |  | TAATGAGTGGGTGGGATGGGT | PCR |
|  | TTTGCTTTTGAGTAACGCCAAGCC | TAATATACAAGGCGCCCGTGG | PCR |
|  |  | TGCAGGAGAAAGAGGTGGGTA | PCR |
|  | TTGc-TTTTGAGTAACGCCAAGC | GGCGAGGGGACACTTCTAAA | PCR |
|  |  | GGCCTCTCTCAAAGAGATGTACC | PCR |
|  | TTTGCTTTTGAGTAACGCCAA-g | GGCGAGGGGACACTTCTAAAT | PCR |
|  |  | TTCTGAAGGCCTCTCTCAAAG | PCR |
| B22 | TTGGTACTCCTTGGCCATGGCGGc | TCCATCCCATTTTTACAGGCAGA | PCR |
|  |  | TACACATTGTTTGGCAGGCAAG | PCR |
|  | TTGGTACTCCTTGGCCATGGCGGc | GAGTCAAGGCTGAGACTGGT | PCR |
|  |  | ATGTGCATGCCAAAATTGGGTTA | PCR |
|  | TTGGTACTCCTTGGCCATGG-cG | GTCCAGAACAGGAAACAAGGC | PCR |
|  |  | GACACTGGACTACCAGAGAGT | PCR |
|  | TTGGTACTCCTTGGCCATGGC-G | GGGAGGAGACTAGACCCACTT | PCR |
|  |  | GCACAGGACATCTTTCCTCCA | PCR |
|  | TTGGTACTCCTTGGCCATGGC-G | GCACCAAAGGCCAAGAACAA | PCR |
|  |  | GGCACATGAGCTGAGTGAAAG | PCR |
